# The eIF4A Inhibitor Silvestrol Blocks the Growth of Human Glioblastoma Cells by Inhibiting AKT/mTOR and ERK1/2 Signaling Pathway

**DOI:** 10.1155/2022/4396316

**Published:** 2022-05-30

**Authors:** Wei Zhang, Pian Gong, Qi Tian, Shoumeng Han, Jianfeng Wang, Peibang He, Yujia Guo, Guijun Wang, Qianxue Chen, Jie Huang, Mingchang Li

**Affiliations:** ^1^Department of Neurosurgery, Renmin Hospital of Wuhan University, Wuhan, 430060 Hubei, China; ^2^Research Center for Stem Cell Engineering and Technology, Institute of Industrial Technology, Chongqing University, Chongqing, China. Better Biotechnology LLC, Chongqing, China

## Abstract

The most frequently identified central nervous system tumor in adults is glioblastoma multiforme (GBM). GBM prognosis remains poor despite multimodal treatment, i.e., surgery and radiation therapy with concurrent temozolomide-based chemotherapy. Silvestrol, an eIF4A inhibitor, has been demonstrated to be able to kill tumor cells in previous studies. In this study, it was found that silvestrol considerably attenuated the proliferative potential of U251 and U87 glioma cells and reduced expression of cyclin D1. In addition, silvestrol reduced the level of ERK1/2 and decreased the levels of AKT phosphorylation. Unfortunately, the effect of silvestrol in inhibiting GBM cells was greatly reduced with hypoxia, and the downregulation in AKT/mTOR and ERK1/2 were also rescued with an upregulation of HIF1*α*, which warranted further research. Taken together, silvestrol exerted antitumor effects in GBM cells by inhibiting the AKT/mTOR and ERK1/2 signaling cascades.

## 1. Introduction

Gliomas are the most prevalent type of primary brain tumor, with 3.19 new cases per 100,000 people each year [1]. Gliomas account for 81% of all central nervous system (CNS) cancers. Gliomas are commonly diagnosed in elderly people, with a median age of 64 at the time of diagnosis. The chances of glioma are 1.5 times higher in men than in women, and white people are approximately two times more at risk of developing brain cancer than nonwhite people [2]. Gliomas are classified into four grades by the World Health Organization (WHO), with grades 1 and 2 gliomas denoting as low-grade glioma (LGG) and grades 3 and 4 gliomas denoting as high-grade glioma (HGG) [3]. The most prevalent form of grade 4 glioma is glioblastoma (GBM) [4]. Patients with newly diagnosed GBM have a median overall survival (OS) of just 12–18 months with the existing therapeutic approaches, including maximal safe resection and external beam radiation, concurrent temozolomide (TMZ), and maintenance chemotherapy [5]. Over the years, several anticancer drugs have been developed, but the FDA has approved only a few of them for glioma treatment. Hence, it is needed to develop candidate drugs against glioma.

It was known that oncogenesis is caused by dysregulated protein translation [6]. Translation initiation is the most important regulated phase in the protein biosynthesis cascade in which the eukaryotic initiation factor 4F (eIF4F) complex is recruited to the 5′ untranslated region (5′ UTR) of mRNA. The complex is made up of three parts, including eIF4E, eIF4A, and eIF4G. eIF4A is an RNA helicase that unfolds the secondary structure of an mRNA's 5′ region [7]. eIF4G is thought to operate as a scaffold for other eIFs and boost eIF4A helicase functioning. Many malignancies have constitutive activation of oncogenic kinases (AKT, mTOR, and ERK1/2) [8, 9]. These kinases block the translational repressors of the eIF4E-binding protein, allowing eIF4F to assemble and result in increased synthesis of proteins. Alternatively, AKT and mTOR may phosphorylate and deactivate an endogenous repressor of eIF4A activity. Squamous lung carcinomas have been found to have eIF4G amplification [10]. Leukemogenesis is accelerated by increased eIF4A expression [11].

Silvestrol has recently gained a lot of attention as an eIF4A inhibitor due to its nanomolar cytotoxic activity against colorectal carcinoma, vestibular schwannoma, and hepatocellular carcinoma [12]. Silvestrol prevents the development of the eIF4F complex by blocking eIF4A1/2. However, there was no study on the anticancer effect of silvestrol in glioblastoma. The reported studies revealed the role of hypoxia in glioma development [13]. Tumor hypoxia is a significant contributor to anticancer therapy failures [14]. Herein, we investigate the role of silvestrol therapy against glioma and its association with hypoxia.

## 2. Materials and Methods

### 2.1. Public Data and Samples Collection and Bioinformatics Analysis

We collected whole-genome RNA-seq expression data and clinical and molecular information from 163 glioma samples in The Cancer Genome Atlas (TCGA) database. The data with incomplete clinical information or lacking prognostic information were eliminated. In addition, 207 normal brain samples with complete mRNA-seq data were used as a control set. According to The Human Protein Atlas (HPA) database, the protein level of eIF4A3 identified among normal brain and glioma tissues was investigated. The prognostic significance of the eIF4A3 in glioma was evaluated by Kaplan–Meier survival analysis in the TCGA-glioma cohort.

### 2.2. Cell Cultures

The Institute of Biochemistry and Cell Biology, Chinese Academy of Sciences (Shanghai, China) provided human GBM cells including U87 and U251. The cell culturing was carried out at 37°C and 5% CO_2_ in DMEM enriched with 10% FBS (Biological Industries) and 1% penicillin/penicillin (Biological Industries), followed by cell culturing in O_2_ (1%), CO_2_ (5%), and N_2_ (94%) in hypoxia condition. In some experiments, cells were exposed to hypoxia and silvestrol simultaneously.

### 2.3. Drugs and Antibodies

Silvestrol was acquired from MCE (Cat.#HY-13251/CS-0543). The dissolution of silvestrol was carried out in dimethyl sulfoxide (DMSO, Beyotime).The following antibodies were used: GAPDH (AF7021, Affinity), HIF1-*α* (NB100-479, Novus Biologicals), p-AKT (1910134,Thermo Fisher), ERK1/2 (NB110-96887, Novus Biologicals), mTOR (WE3267992E, Invitrogen), cleaved caspase-3 (Ab32042, Abcam), eIF4A3 (WD3239677D, Invitrogen), cyclin D1 (AF0931, Affinity), Alexa Fluor™ ^488^ goat anti-rabbit IgG (A11008, Invitrogen), Alexa Fluor™ ^594^ goat anti-rabbit IgG (A11012, Invitrogen), HRP AffiniPure Goat Anti-Rabbit IgG (E030120, Earthox).

### 2.4. Clinical Samples

Glioma tissues were obtained from the Department of Neurosurgery in Renmin Hospital of Wuhan University from June 2016 to June 2019.A total of 20 paraffin-embedded glioma tissue were used for immunofluorescence staining. All patients signed informed consents, and this study received the approval of the Ethics Committee of Renmin Hospital of Wuhan University.

### 2.5. Cell Viability Assay

The MTT test was carried out to evaluate the anticancer activity of silvestrol, as suggested by the manufacturer (Beyotime, Shanghai, China). The culturing of U87 and U251 cells (5,000 cells/well) was carried out in 96-well plates. After adhering and reaching the logarithmic growth phase, the cells were exposed to various concentrations of silvestrol (0, 5, 10, 20, 50, 75, 100, 200, 300, 400, or 500 nM) for one or two days. Each sample received 40 *μ*L of MTT solution at each time point, followed by adding DMSO (100 *μ*L) after 4 hours of incubation at 37°C to dissolve the formazan crystals. A microplate reader was used to detect light absorbance at a wavelength of 570 nM. To alter the final optical density (OD) values, the absorbance of the medium without cell culture was adjusted. Four different assays were performed on each group.

### 2.6. Western Blotting

After being incubated with 0, 10, or 20 nM silvestrol for 24 hours, the U87 and U251 cells were thoroughly rinsed (twice) with PBS and then lysed on ice for 0.5 hours with RIPA Lysis Buffer (Beyotime, China), followed by centrifugation (1.4 × 10^5^ rpm) for 15 minutes. The BCA technique was used to evaluate the protein concentration (Beyotime, China), followed by adding the lysate with a specific ratio of 5∗ loading buffer (Solarbio) and boiling for 10 minutes at 100°C. Next, 8% or 12% SDS-PAGE was conducted to separate an equal amount of proteins, followed by moving onto PVDF membranes (Biosharp). Nonfat milk (5%) was used to block PVDF membranes for 60 min at room temperature before being incubated at 4°C with primary antibodies (p-AKT, AKT, and cyclin D1) for 24 hrs at the dilution ratio specified by the supplier. After that, the membranes were incubated for 60 min with secondary antibodies (labeled with HRP). The imaging system (AMERSHAM ImageQuant 800) was employed to analyze the results.

### 2.7. Immunofluorescence Staining

The cells fixation was carried out with paraformaldehyde (4%) for 15 min, followed by penetration with 0.5% Triton X-100 solution in PBS at ∼25°C for 10 min. Next, the slides were rinsed three times with PBS. After 60 min at room temperature, immunol staining blocking buffer (Beyotime) was added dropwise to the slides, which were then incubated with the diluted primary antibodies for 24 hours at 4°C in a moist box, followed by incubating with the secondary antibodies (Antgene, Wuhan, China) for 60 min at 37°C in the dark. Staining was performed by adding DAPI (ANT046, Antgene) for 5 min in the dark, followed by examining the slides under a fluorescence microscope (Leica, Germany).

Three-micron slices were cut and placed on coated glass slides and then baked for two hours at 60°C in a vertical position, followed by dewaxing with xylene, rinsing with a graded alcohol series, and rehydration with distilled water. Antigen retrieval (AR) was carried out using a citrate antigen retrieval solution (Beyotime). Blocking buffer for immunol staining (Beyotime) was placed dropwise on the slides at room temperature for 1-hour postcooling of sections to roughly 50°C. Following that, the incubation was performed with the diluted primary antibodies for 24 hours at 4°C in a moist box, followed by incubating with the secondary antibody for 60 min at 37°C in the dark. Furthermore, staining was performed by adding DAPI (ANT046, Antgene) for 5 min (in the dark), followed by examining the slides under a fluorescence microscope.

### 2.8. Statistical Evaluations

SPSS 20 was employed to evaluate the statistical data. The obtained data is presented as a mean ± SD. *P* value <0.05 was regarded as statistically significant. The two-tailed Student's *t*-test and ANOVA were carried out to evaluate the variations.

## 3. Result

### 3.1. The Expression of eIF4A3 and Its Association with Prognosis in the TCGA Dataset

**Figure 1:**
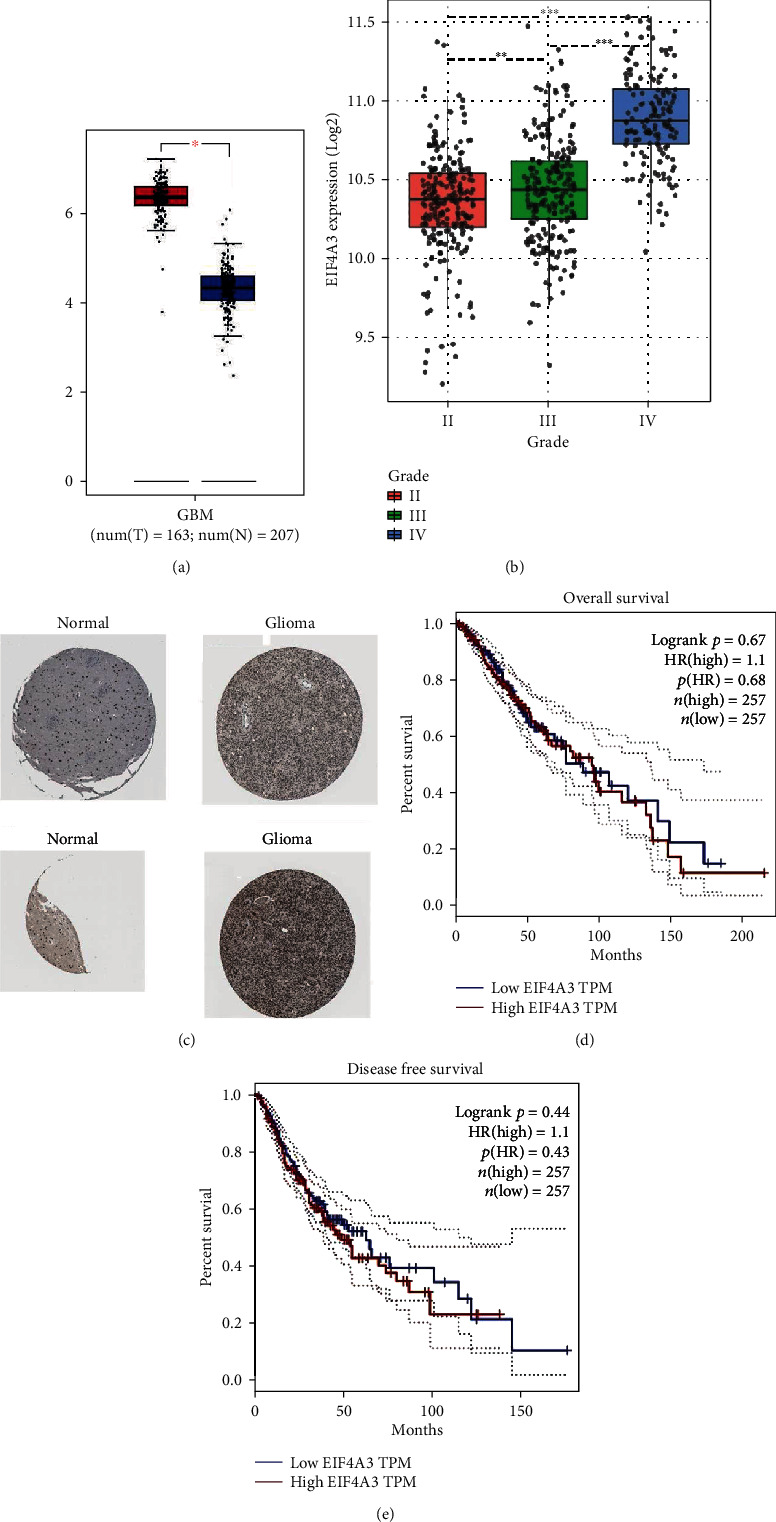
(a) Expression of eIF4A3 in glioma patients and non-diseased population in TCGA. (b) Expression level of eIF4A3 in glioma patients stratified by WHO grade in TCGA. (c) The expression of eIF4A3 protein in normal and glioma tissues according to the HPA database. (d, e) Survival analysis with eIF4A3 in glioma patients in TCGA.

We analyzed the expression differences of eIF4A3 in nontumor tissues and glioma tissues of various grades and analyzed the relationship between eIF4A3 expression and glioma prognosis in the TCGA dataset. The expression of eIF4A3 in tumor tissues was significantly higher than that in nontumor tissues. Additionally, the expression of eIF4A3 increased with the increasing grade of glioma. However, the expression of eIF4A3 was not significantly associated with prognosis in glioma patients (Figure 1).

### 3.2. The Expression of eIF4A3 Is Associated with Glioma Grade in Tissue Samples

**Figure 2:**
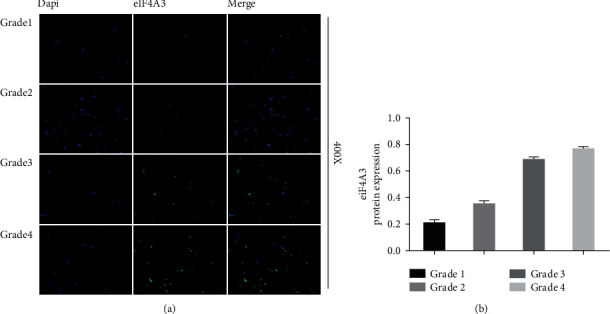
(a) eIF4A3 express more with the degree of malignancy increases in glioma (defined by WHO Grade). Shown are representative immunofluorescence images in glioma for eIF4A3. (b) Immunofluorescence analysis of eIF4A3 in glioma tissues of various grades.

The tissue samples obtained from gliomas of various grades were immunostained to analyze the eIF4A3 expression levels. It was found that the higher the grade of glioma, the higher the expression of eIF4A3. Furthermore, eIF4A3 was rare in glioma of grade 1 but abundantly expressed in gliomas of grade 4 (Figure 2).  Table 1 summarizes the comparative staining signals from each sample. This result was consistent with that obtain from the TCGA dataset.  Table 2 shows the clinical information of 20 patients in this study.

### 3.3. Silvestrol Inhibits the Proliferative Ability of Human GBM Cells

**Figure 3:**
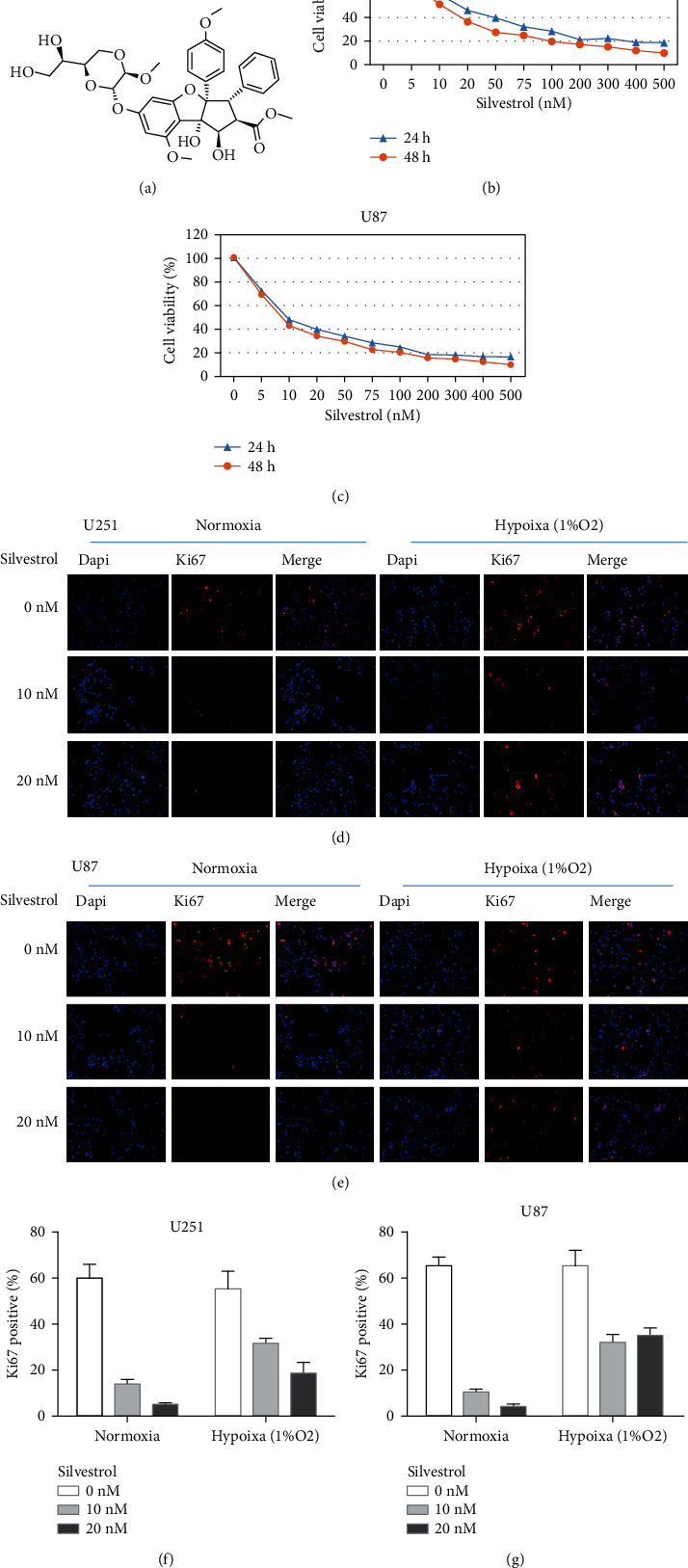
Silvestrol decreases the proliferative potential of the human GBM cells. (a) The molecular structure of silvestrol. (b, c) After being exposed to different concentration of silvestrol (0, 5, 10, 20, 50, 75, 100, 200, 300, 400, and 500 nmol/L) for 24 or 48 hours, the viability of U251 and U87 cells was assessed using the MTT assay. The IC50 values of silvestrol for U251 and U87 cells were found to be 22.883 nmol/L and 13.152 nmol/L (24 hours). (d, e) We used Ki67 to evaluate the proliferation ability of GBM cells by immunofluorescence. The proliferative capacity of GBM cells decreased significantly with silvestrol treatment, but it was rescued by hypoxia. (f, g) Immunofluorescence analysis of Ki67 in U251 and U87 cells treated with silvestrol and hypoxia.

The chemical structure of the eIF4A inhibitor, silvestrol, is shown in Figure 3(a). MTT assays was performed to assess if this inhibitor had any effect on GBM cells. It was found that the proliferation of U87 and U251 cells was potently inhibited by silvestrol. Moreover, the silvestrol effect on cell viability was concentration-dependent (Figures 3(b) and 3(c)). We observed that the IC50 values of silvestrol for U251 and U87 cells were 22.883 nmol/L and 13.152 nmol/L (24 hours), respectively. Ki67 assay of immunofluorescence indicated that silvestrol can inhibit GBM cells proliferation, but this effect was significantly reduced in hypoxic conditions (Figures 3(d) and 3(e)). Based on the above results, silvestrol exhibited antiglioma property, and this property was weakened by hypoxia.

### 3.4. Silvestrol Inhibits the Expression of Cyclin D1

**Figure 4:**
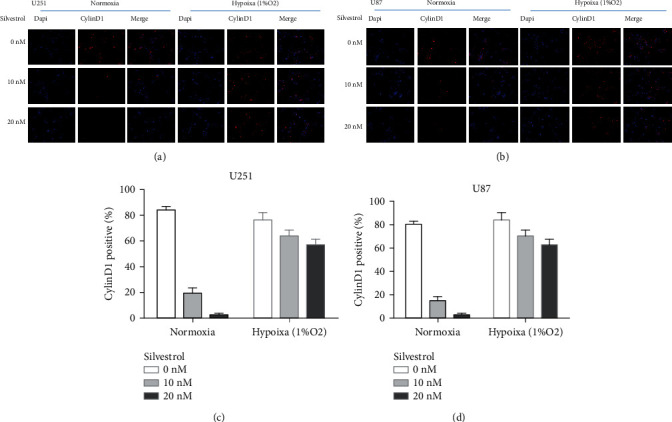
(a, b) We detected changes in cyclin D1 with silvestrol treatment and hypoxia treatment for 24 hours by immunofluorescence. We found that cyclin D1 was obviously suppressed, but this inhibitory effect was significantly reduced by hypoxia. (c, d) Immunofluorescence analysis of cyclin D1 in U251 and U87 cells treated with silvestrol and hypoxia.

We investigated the expression of cyclin D1 protein, a crucial checkpoint in the cell cycle, to explore the mechanism of silvestrol-induced cell cycle arrest (Figure 4). The U87 and U251 cells were exposed to hypoxia or varying doses of silvestrol. Compared with the control group, cyclin D1 was obviously suppressed in immunofluorescence. But this inhibitory effect was significantly reduced in hypoxic conditions.

### 3.5. The AKT/mTOR and ERK1/2 Signaling Cascade Is Blocked by Silvestrol in U87 and U251 Cells

**Figure 5:**
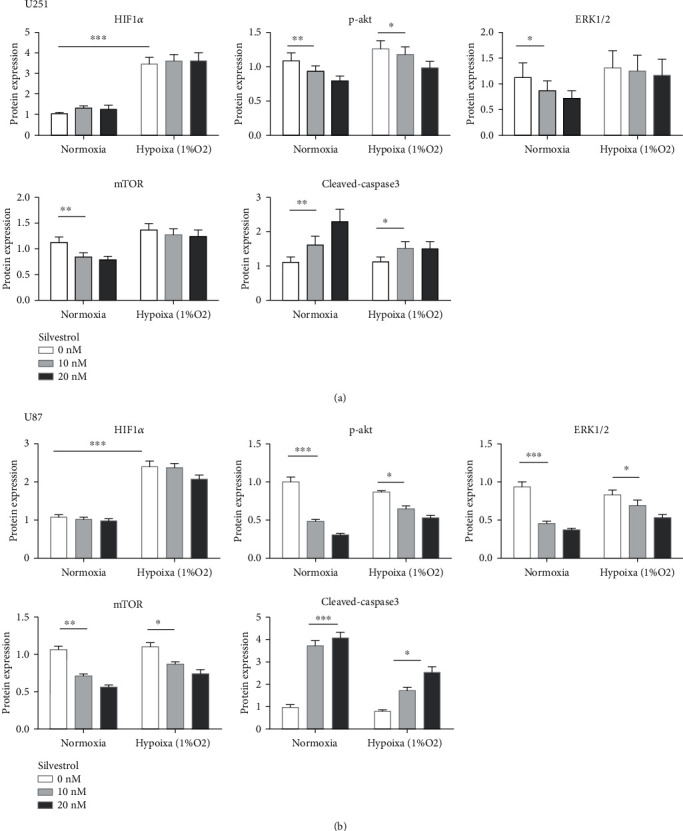
(a, b) Expression of the HIF1*α*, p-AKT, ERK1/2, mTOR, and cleaved caspase-3 in U251 and U87 cells exposed to various concentrations of silvestrol (0, 10, and 20 nmol/L) and hypoxia for 24 hours. GAPDH was used for normalization. (c, d) Western blot analysis of HIF1*α*, p-AKT, ERK1/2, mTOR, and cleaved caspase-3 levels in U251 and U87 cells treated with silvestrol and hypoxia.

Silvestrol revealed a potent antitumor activity on glioma cells, which prompts us to explore its underlying mechanism. First of all, we found that HIF1-*α* increased significantly with hypoxic treatment, and this change had no association with silvestrol. p-AKT decreased obviously in the cells treated by silvestrol, but the decrease was rescued with hypoxic treatment. For the downstream target of p-AKT, mTOR, we observed a similar phenomenon to that of p-AKT. Interestingly, we found a low expression of ERK1/2 with the treatment of silvestrol, and it was rescued by hypoxia. We also evaluated apoptosis by the expression of cleaved caspase-3, a classic apoptotic protein. Whether under hypoxic condition or normoxic condition, we observed an increase of cleaved caspase-3 with treatment of silvestrol, but the increase under hypoxic conditions was not as significant as the other (Figure 5). These results suggested that the inhibitory activity of silvestrol against glioma cell proliferation is most likely due to its modulatory effect on the AKT/mTOR and ERK1/2 signaling cascades.

## 4. Discussion

Gliomas are one of the most often diagnosed brain tumors, with more than half of them being GBMs, the most malignant type of CNS tumor. Even with the largest feasible surgical resection, the median survival time of patients receiving radiotherapy and chemotherapy is less than 15 months. Recent studies have shown that eIF4A3 is significantly upregulated in several malignant tumors, such as hepatocellular carcinoma, pancreatic cancer, and ovarian cancer [15]. The level of eIF4A3 in glioma was significantly higher than that in nontumor tissue, and the level of eIF4A3 increased with the grade of glioma. However, the expression of eIF4A3 had no obvious effect on the prognosis of glioma patients, which may be related to the poor prognosis of glioma patients.

It was also found that eIF4A3 is expressed at low level in LGG and high level in HGG in tissue specimens of gliomas, which suggests that eIF4A3 may be related to the malignant behavior of gliomas. Additionally, our research has demonstrated that silvestrol, an eIF4A inhibitor, can inhibit the proliferation of gliomas and promote their apoptosis in vitro by MTT assay. These results suggest that silvestrol may be a potential drug for the treatment of glioma. Oblinger et al. have reported that silvestrol can inhibit vestibular schwannoma through the AKT pathway and inhibit meningiomas through the AKT and ERK1/2 pathway [16]. The serine/threonine protein kinase Akt is activated in a variety of human tumors and is the central link in the PI3K/AKT/mTOR signaling pathway [17]. Akt plays an important role in regulating tumor cell growth and proliferation, promoting cell invasion and metastasis, and promoting angiogenesis and tumor cell resistance to chemotherapy and radiotherapy. A serine/threonine-protein kinase is an extracellular signal-regulated kinase (ERK). Its aberrant activation has a major impact on the onset and progression of cancers as it is a major downstream protein in the RAS/RAF/MEK/ERK signaling cascade [18]. In the present study, we have confirmed that silvestrol can inhibit glioma through the AKT/mTOR and ERK1/2 signaling pathway.

Hypoxic environment is the basic feature of glioma malignancy. The aggressive clinical behavior of gliomas is closely related to the oxygen in the tumor microenvironment [19]. The tumor microenvironment has been extensively researched for its role in promoting tumor growth and clinical therapy efficacy [20]. Hypoxia is a common hallmark of highly aggressive and fast-growing tumors due to limited or abnormal blood flow. Cancer cells activate numerous biological pathways in response to hypoxia, which contributes to treatment resistance and cancer progression. In gliomas, hypoxia is closely related to temozolomide resistance and radiotherapy resistance [20]. Therefore, in this study, we also have explored whether hypoxia has an effect on the resistance of silvestrol. It was found that the inhibitory effect of silvestrol on glioma cells is significantly weakened when the glioma cells were in the hypoxic condition. Under normoxia, HIF1*α* will be degraded by E3 ubiquitin ligase. Under hypoxic conditions, HIF1*α* will accumulate, transfer to the nucleus, and bind to hypoxia response element (HRE), thereby activating the transcription of target genes [21]. In our study, it was found that HIF1*α* of glioma cells under hypoxia treatment entered the nucleus and the expression increased significantly, and the changes of AKT, mTOR, and ERK1/2 under silvestrol treatment were rescued. Therefore, we speculate that the effect of silvestrol in inhibiting glioma may be an oxygen-dependent and related to HIF1*α*.

The poor therapeutic effect of glioma is also closely related to the blood-brain barrier [22]. But unfortunately, there is no literature showing that silvestrol can pass through the blood-brain barrier, which poses a challenge for further clinical application and urges us to further conduct in situ tumorigenesis experiments to verify its effectiveness.

In summary, we have found that silvestrol exerted its antiglioma effect in vitro mainly through the AKT/mTOR and ERK1/2 signaling pathways. However, glioma cells are resistant to silvestrol under hypoxic condition which is related to HIF1*α*. Our results suggest that silvestrol is a potential drug for the treatment of glioma.

## Figures and Tables

**Table 1:** Immunofluorescence staining signals for eIF4A3 in gliomas of various grades. Tissue sections were processed using immunohistochemistry analysis. Visual inspection of the intensity and percentage of immunopositive cells yielded a 0 to 3+ scale for quantifying the immunofluorescence signal. In this table, 0, 0.5, 1, 2, 3, and 3+ have been indicated as negative, weak positive, moderate positive, strong positive, very strong positive, and full of vision, respectively.

ID no.	Glioma grade	eIF4A3
1	1	0.5
2	1	1
3	1	1
4	1	1+ (occasional 2)
5	1	0 (occasional 0.5)
6	2	2
7	2	2
8	2	2
9	2	2
10	2	1+ (occasional 2)
11	3	2
12	3	2-3
13	3	1+ (occasional 2)
14	3	3
15	3	2
16	4	2+(occasional 3)
17	4	3
18	4	3+
19	4	2
20	4	2

**Table 2:** Clinical information of the patients in this study.

Patient	Sex	Age (y)	Location	Pathological type	WHO grade
1	M	21	Cerebellum	Pilocytic astrocytoma	I
2	M	8	Cerebellum	Pilocytic astrocytoma	I
3	M	15	Cerebellum	Pilocytic astrocytoma	I
4	M	31	Sellar region	Capillary hemangioblastoma	I
5	M	18	Cerebellum	Pilocytic astrocytoma	I
6	F	37	Insula	Diffuse astrocytic glioma	II
7	M	37	Frontal lobe	Oligodendroglioma	II
8	M	50	Frontal lobe	Gemistocytic astrocytoma	II
9	F	68	Frontal lobe	Oligodendroglioma	II
10	M	64	Frontal lobe	Oligodendroglioma	II
11	M	43	Frontal lobe	Anaplastic astrocytoma	III
12	M	60	Frontal lobe	Anaplastic astrocytoma	III
13	M	63	Temporal lobe	Anaplastic astrocytoma	III
14	M	32	Frontal lobe	Anaplastic oligodendroglioma	III
15	F	49	Temporal lobe	Anaplastic astrocytoma	III
16	M	49	Parietal lobe	Glioblastoma	IV
17	M	65	Parietal lobe	Glioblastoma	IV
18	F	58	Temporal lobe	Glioblastoma	IV
19	F	57	Temporal lobe	Glioblastoma	IV
20	F	34	Temporal lobe	Glioblastoma	IV

## Data Availability

Emails could be sent to the address below to obtain the shared data: mingcli@whu.edu.cn.
